# MRI combined with PET-CT of different tracers to improve the accuracy of glioma diagnosis: a systematic review and meta-analysis

**DOI:** 10.1007/s10143-017-0906-0

**Published:** 2017-09-16

**Authors:** Yihan Yang, Mike Z. He, Tao Li, Xuejun Yang

**Affiliations:** 1 Department of Neurosurgery, Tianjin Medical University General Hospital, No. 154 Anshan Road, Heping District, Tianjin, 300052 China; 2 Columbia University Mailman School of Public Health, New York, NY USA

**Keywords:** Glioma, MRI, PET-CT, Diagnostic accuracy

## Abstract

Based on studies focusing on positron emission tomography (PET)-computed tomography (CT) combined with magnetic resonance imaging (MRI) in the diagnosis of glioma, we conducted a systematic review and meta-analysis evaluating the pros and cons and the accuracy of different examinations. PubMed and Cochrane Library were searched. The search was conducted until April 2017. Two reviewers independently conducted the literature search according to the criteria set initially. Based on the exclusion criteria, 15 articles are included in this study. Of all studies that used MRI examination, there are five involving 18F-fluorodeoxyglucose-PET, five involving 11C-methionine-PET, five involving 18F-fluoro-ethyl*-*tyrosine-PET, and three involving 18F-fluorothymidine-PET. Due to the limitations such as lack of data, small sample size, and unrepresentative studies, we use a non-quantitative methodology. MRI examination can provide the anatomy information of glioma more clearly. PET-CT examinations based on tumor metabolism using different tracers have more advantages in determining the degree of glioma malignancy and boundaries. However, information provided by PET-CT of different tracers is not the same. With respect to the novel hybrid MRI/PET examination equipment proposed in recent years, the combination of MRI and PET-CT can definitively improve the diagnostic accuracy of glioma.

## Introduction

Glioma is one type of intracranial space-occupying lesions with relatively high incidence [51, 55, 80]. In general, the management of glioma consists of imaging, surgery, and other postoperative treatment modalities [34, 38]. In terms of surgical options, including the selection of tumor resection or stereotactic biopsy, the determination of tumor margins during surgery, the necessity of postoperative radiation therapy, or even the decision of the radio-therapeutic modalities, preoperative imaging studies can provide crucial information [21]. As imaging technology continues to develop, a variety of different imaging methods are appearing, and a one imaging modality is becoming difficult to meet clinical needs. The concept of multi-modal imaging was therefore introduced, utilizing the information of two or more medical imaging modalities combined together to obtain more abundant and accurate information about a disease [6].

In actual clinical practice, conventional magnetic resonance imaging (MRI) examination is the first test for patients with suspected glioma. An MRI provides preliminary information about the tumor, including tumor location, size, and boundaries [25, 63, 77]. Information from a high-quality MRI image can be used in an operative setting and can provide valuable information to the surgeons. This information will improve the quality of surgery, closely related to the prognosis [79]. Furthermore, it can effectively assist the accurate grading of glioma [13]. Despite these advantages, diagnostic information provided by conventional MRI is preliminary and has a number of shortcomings, such as the lack of effectiveness under the absence of blood-brain barrier damage and difficulty in identifying abnormal imaging as tumor recurrence (tumor progression) or pseudo-progression [77].

In order to compensate the drawbacks of conventional MRI examination as mentioned above, positron emission tomography (PET)-(computed tomography) CT, based in tumor metabolic imaging, is used as a further test for suspected glioma [46]. PET-CT imaging is of vital importance of the functional imaging in glioma diagnosis. Combined with the anatomy information given by conventional MRI, PET-CT provides an important basis for more sensitive glioma treatment [60]. For example, when cases are difficult for MRI to evaluate, PET-CT can provide important notes for the diagnosis and imaging data to develop plans for the operation. In addition, it also provides indispensable information for patients’ prognoses [7, 19, 30, 32, 36, 67, 85].

This paper summarizes research in the current literature that uses multi-modal images (conventional MRI and PET-CT) for the diagnosis of glioma. It then systematically categorizes search results to obtain a comprehensive conclusion, providing valuable information for the diagnosis, treatment, and prognosis of different graded gliomas, concurrently serving as a foundation for future researchers interested in multi-modal imaging of glioma diagnosis.

## Materials and methods

Using PubMed and Cochrane Library, two reviewers independently conducted the literature search. We temporarily ignore the restrictions of language in the document retrieval process. Initial inclusion criteria included (1) researches on the diagnostic imaging of glioma and (2) imaging methods must include PET-CT regardless of the type of molecular reagent; at the same time, study must also involve the use of MRI imaging.

After the initial screening, reviewers detailedly reviewed articles from the initial screening and excluded articles using six exclusion criteria. Exclusion criteria included (1) sample size less than 10; (2) no comparison of two kinds of imaging methods; (3) research does not aim at improving the accuracy of glioma diagnosis, for example, research focused on the prognostic assessment; (4) study does not involve conventional MRI but advanced MRI, for example MRS and so on; (5) non-clinical studies, including animal level and cellular level research; and (6) non-English literature.

**Fig. 1:**
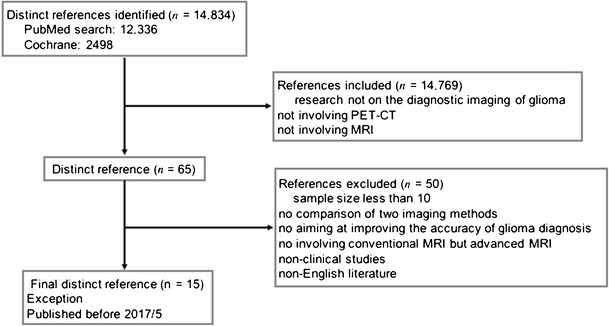
Flow diagram of the study selection process

After the above screening, results obtained by the two reviewers were compared. Any disagreements were given to a third reviewer to determine whether the disputed article should be included in the systematic review (Fig. 1).

After the search in accordance with the above criteria, only a limited number of articles were found. Furthermore, samples of relevant studies are heterogeneous, and internal limitations cannot be ignored. Therefore, studies that merely aim at a particular glioma sub-category, such as research involving only high-grade gliomas, were not excluded. Studies that took place immediately after a specific situation, such as glioma recurrence after surgery and glioma assessment before radiotherapy, were also not excluded. Due to these limitations, the compiled data cannot be analyzed using traditional statistical methods. Instead, we use a non-quantitative methodology as our primary evaluation system, which compiles effective information from each article that is then further analyzed and discussed.

The two researchers extracted information from each of the documents independently. The information required includes the main topics of each study, the subjects studied, the approaches of data collection and integration, the conclusions, and the core information discussed in the results. After that, the researchers synthesize and contrast the extracted information.

## Results

### Included studies

After the initial screening, 12,336 articles were found in PubMed and 2498 articles were found in Cochrane Library. After reading the titles and abstracts of these articles, we retrieved a total 65 articles involving one or more of PET-CT examination combined with conventional MRI. Based on exclusion criteria, 15 articles are included in this study. According to the different PET-CT tracer, there are five studies involving 18F-fluorodeoxyglucose (FDG)-PET, five studies 11C-methionine (MET)-PET, five studies involving O-(2-[18F]fluoroethyl)-L-tyrosine (18F-FET)-PET, and three studies involving 3’-[18F]fluoro-3’-deoxythymidine (18F-FLT)-PET (Table 1). Coincidentally, these four different PET-CT tracers are the four types frequently used clinically.

**Table 1:** Included studies

Authors and year	PET tracer	Subjects	Glioma type	Patients received therapy before
Bšelohlávek et al., 2002	18F-FDG	29	LGG, HGG	+
Pauleit et al., 2005	18F-FET	28	WHO grade I~IV	–
Walter et al., 2005	18F-FET	45	WHO grade I~IV	+
Pirotte et al., 2006	11C-MET, 18F-FDG	103	LGG, HGG	–
Mira et al., 2004	11C-MET	10	GBM	–
Yamamoto et al., 2006	18F-FLT	10	GBM (recurrent)	+
Galldiks et al., 2010	11C-MET	12	Glioblastoma	+
Ewelt et al., 2011	18F-FET	30	WHO grade II~IV	–
Santra et al., 2012	18F-FDG	90	WHO grade I~IV	+
Arbizu et al., 2012	11C-MET	23	WHO grade II~IV	+/−
Jansen et al., 2012	18F-FET	127	WHO grade I~IV	–
Singhal et al., 2012	11C-MET, 18F-FDG	102	WHO grade I~IV	NA
Nowosielski et al., 2014	18F-FET, 18F-FLT	23	WHO grade III, IV	–
Zhao et al., 2015	18F-FLT	19	WHO grade III, IV	+
Song et al., 2016	18F-FDG	70	NA	–

*NA* = not available

### Data of researches

When analyzing the data from the researches (Table 2), we can see that, in general, although specificity of conventional MRI is relatively low, it has a relatively higher sensitivity. On the contrary, the sensitivity in the majority of PET-CT is not ideal, but the specificity is relatively high. When we make a comparison between low-grade gliomas and high-grade gliomas, we find that in terms of relatively low-grade gliomas, PET-CT for the diagnosis of high-grade gliomas indeed has an advantage, a point that coincides with our clinical experience. In terms of other articles and other data with heterogeneity, our analysis will be described in detail below.

**Table 2:** Data of researches

		Average sensitivity/SD	Average specificity/SD	Average accuracy/SD
MRI	Overall	95.5%/0.99%	90.05%/6.75%	74.9%/8.38%
	LGG	67.57%/42.38%	26.2%/7.56%	63%/4%
	HGG	82.67%/14.41%	50.2%/29.96%	76.5%/9.5%
18F-FDG-PET	Overall	66%/3.5%	90.05%/6.75%	70.9%/6.44%
	LGG	65%/25%	100%/0	81%/14%
	HGG	63.2%/9.42%	88.87%/7.87%	66.75%/4.25%
11C-MET-PET		NA/NA	NA/NA	72%/NA
18F-FET-PET	Overall	92%/NA	81%/NA	NA/NA
	LGG	66.35%/12.55%	11.8%/NA	NA/NA
	HGG	93.05%/4.85%	46.1%/NA	NA/NA
18F-FLT-PET		NA/NA	NA/NA	NA/NA

*SD* = standard deviation; *LGG* = low-grade glioma; *HGG* = high-grade glioma; *NA* = not available

## Discussion

Glioma is the most common primary type of the nervous system tumors with its own unique heterogeneity. Depending on the characteristics of growth patterns and so on, glioma can be subclassed into WHO grade I, II, III, and IV. Overall, it can fall roughly into two categories, namely low-grade gliomas (LGG) and high-grade gliomas (HGG). From the perspective of growth pattern, low-grade gliomas grow relatively quietly, while high-grade gliomas grow actively, showing a kind of “aggressive” growth mode [53]. Furthermore, glioma is a typical heterogeneous tumor [42, 53]. In the same tumor, there may be different grades of glioma components. This poses a greater problem before glioma surgery, including the development of postoperative treatment plans before radiotherapy. Thus, the use of a single imaging means inevitably cannot meet the needs of clinical practice. And glioma diagnosis must therefore be with the support of multi-modality image fusion technology.

### Conventional MRI

As a better way of presenting intracranial structures than CT or other imaging scan, conventional MRI is used as the basic examination methods. It is the indispensable cornerstone of a variety of imaging means [61]. Published studies suggest that if surgery were done to remove 98% of disease displayed by enhanced MRI, the survival of glioblastomas would be benefited. In low-grade gliomas, if expansion resection was based on the T2-weighted MRI sequences, it is possible to predict overall survival [39, 65, 72]. However, conventional MRI judgment indeed has significant limitations when deciding glioma invasion and boundaries. For example, in anaplastic gliomas, MRI may show no contrast enhancement, and neoplastic cells often occur in regions outside the abnormal signal intensity. PET imaging may provide useful information in such situations [56, 64, 75]. So when (1) during the planning stages before glioma surgery or (2) postsurgery but before radiotherapy, PET-CT findings usually are added to improve the accuracy of tumor margin determination during the surgical removal process. 18F-FDG-PET and 11C-MET-PET are now commonly used in clinical PET examination [49]. In addition, 18F-FET-PET and 18F-FLT-PET are also applied. A combination of both MRI and PET-CT can provide more complete information, including the scope of the target tumor, thus providing a reliable basis for radiotherapy and neuro-navigation surgery before treatment program development.

### 18F-FDG-PET versus conventional MRI

The action principle of 18F-FDG-PET is that 18F-FDG reagent can be uptaken by normal brain tissue and intracranial neoplastic tissue and be phosphorylated. But it cannot complete the normal glycolytic pathway, so it remains in cells. For intracranial tumor tissue, glucose utilization of tumor cells is relatively increased compared to normal brain tissue. At the same time, their metabolism also transforms from aerobic to anaerobic [45]. This behavior has a great relevance to the grade of gliomas. Thus, 18F-FDG-PET can be used as a means of judging the degree of malignancy. Furthermore, there is no uptake of 18F-FDG in the necrotic area, which can be effectively identified by 18F-FDG-PET. However, 18F-FDG-PET also has its own limitations. First, the presence of normal gray matter will also show increased 18F-FDG uptake. In addition, with the exception of higher grade of glioma, other types of glioma remain iso-metabolic or hypo-metabolic compared to adjacent normal gray matter, which makes it difficult to distinguish between glioma tissue and normal brain tissue by using.

According to the literature we reviewed in this study, 18F-FDG PET-CT is superior in the diagnosis of gliomas to conventional MRI. Additionally, 18F-FDG PET-CT has some advantages in estimating glioma grade. In some cases of low-grade gliomas showing contrast-enhanced image in enhanced MRI, 18F-FDG-PET examinations will show low 18F-FDG uptake [71]. In cases suspected of tumor recurrence after treatment, including recurrent WHO grade II glioma conversion to the anaplastic glioma, early diagnosis and treatment are particularly critical for a good prognosis [15, 50]. And in cases like this, due to necrosis after treatment or necrosis happening simultaneously with tumor progression leading to the destruction of the blood-brain barrier (BBB) and the enhanced MRI diagnosing brain tumors, the diagnosis reliability of recurrence of enhanced MRI after treatment may be reduced [16, 59, 78]. Studies have shown that 18F-FDG-PET for the detection of tumor recurrence has objectively high sensitivity and high accuracy. In addition, for patients having experienced surgery treatment doubted of glioma recurrence, abnormal signal in a conventional MRI may show a tumor lesion or may also be a reaction after treatment (such as radiation necrosis). At this time, 18F-FDG-PET can effectively distinguish which part is metabolic activity and which is tissue necrosis, which in turn provides information to infer the actual tumor size [66]. From another perspective, this shows that although 18F-FDG-PET has its advantages, conventional MRI examination is the essential foundation for inspection. Due to the relatively low 18F-FDG-PET’s sensitivity, it is not as the first step in the diagnosis of glioma patients whether or not the tumor is a recurrence. For MRI results that found abnormalities, then the 18F-FDG-PET will have a certain advantage. In addition, various characteristics of 18F-FDG-PET make it possible for it to become a tool of judging glioma prognosis [11, 12]. Another point worthy of note is that studies suggest there is no obvious link between PET lesion volume and survival in patients. The intensity of FDG uptake of tumor may instead be a more powerful predictor of survival [52].

### 11C-MET-PET versus MRI

11C-MET-PET imaging is based on the needs of the cell protein synthesis precursors, which in turn is related to the proliferation of tissues and the degree of malignancy [9, 14, 49]. Since tumor tissue cells, compared with adjacent normal tissue cells, contain a richer metabolism-related protein, 11C-MET uptake will be richer. As a result, clear tumor boundaries against adjacent normal brain tissue will be formed [8, 33, 44, 76]. Furthermore, comparative 11C-MET-PET and MRI studies have shown that 11C-MET-PET can more effectively show the actual boundaries of the tumor (tumor range) [23, 37]. Regardless, 11C-MET-PET also has its shortcomings, mainly having only a half-life of 20 min, which is relatively short [61].

For enhanced MRI, especially in diagnosing glioblastoma multiforme (GBM), including recurrent GBM, the extension of tumor cells would exceed the scope of gadolinium developing. In many situations, 11C-MET-PET can form a tumor range larger than Gd does. The larger of the actual tumor diameter, the bigger the discrepancy between 11C-MET-PET and enhanced MRI there will be [43]. Existing studies have shown that (1) the tumor margin of 11C-MET-PET is larger than that of enhanced MRI and (2) there are scenarios where the conclusion of enhanced MRI is radiation necrosis but 11C-MET-PET concludes that there is a tumor. In both scenarios above, 11C-MET-PET provides the correct diagnoses upon verification by pathological examination. For most of the GBM, the target range of MRI is shown within the range of PET, so the tumor margin in the surgery is determined by 11C-MET-PET [2]. In the T2 sequence, T2-high area will be larger than the 11C-MET-PET area in the majority of cases. But there are some cases where tumor ranges of 11C-MET-PET displaying in some areas more than that of T2-high areas, indicating that the actual margin of GBM remains beyond T2-high area. T2-high area exceeding 11C-MET-PET displaying range is considered as peritumoral edema. In low-grade gliomas, the tumor range depicted by T2-weighted MRI is larger than the display range of 11C-MET-PET. When selective biopsies were done in these areas, no tumor tissue in these regions or less aggressive lesions were found. Therefore, in cases when T2-weighted MRI is difficult to determine the tumor border, 11C-MET-PET can be used as tool to determine the boundary of tumor for tumor resection [2, 24, 35, 48, 70, 73]. 11C-MET-PET is a powerful means of detecting low-grade gliomas, which may provide useful information for surgery and stereotactic biopsy planning.

### 18F-FET-PET versus MRI

In 18F-FET-PET, tumor tissue and endothelial cell specifically uptake the tracer, and the uptake volume depends on the number of cell density and tumor microvessel density [74]. Some factors can increase BBB damage, which also leads to increased uptake of amino acid tracers [18, 26, 40, 57]. 18F-FET can be produced in mass (18F-labeled amino acid) and it can meet daily clinical needs [83]. In human plasma, 18F-FET will not decrease because of metabolism, and it has good stability in the tumor tissue inside the brain and brain tissue itself within 15 min after injection. It is possible for 18F-FET-PET in clinical practice to provide important information of brain lesion discovering, prognosis assessment, and tumor grading [18–20, 54, 58, 81].

Literature from our review shows that 18F-FET-PET can identify high-grade gliomas that enhanced MR imaging is unable to detect. 18F-FET-PET in conjunction with conventional MRI is often able to achieve higher diagnostic accuracy for glioma diagnosis, while MRI alone is difficult to achieve the same result [47]. Interestingly, Nowosielski et al.’s study showed that a considerable portion of 18F-FET uptake range greater than the range of enhancement of MRI displaying, and the uptake of 18F-FET has only a moderate correlation with contrast volume in enhanced MRI. On the other hand, Christian Ewelt et al. found that 18F-FET uptake always falls within the scope of MRI abnormalities’ signal range in WHO grade II to grade IV gliomas. More research should be carried out on this topic in the future. In cases suspected low-grade gliomas after MRI scan, the positive 18F-FET-PET tends to have higher possibilities of indicating tumor correctly. Note that if it revealed a kinetic but not conventional analysis of 18F-FET uptake after 18F-FET-PET scan, the tumor may be high-grade gliomas [29]. Experiments show that if the result of 18F-FET-PET is negative, then the possibility come to a diagnosis of malignant glioma is not high [17].

### 18F-FLT-PET versus MRI

In the 18F-FLT-PET imaging process, 18F-FLT move through the cell membrane into the cell by facilitated diffusion. It is then phosphorylated, assisted by TK1, to present intracellular trapping. During DNA synthesis, TK1 increased tenfold. In the same time, due to the more active proliferation behavior, glioma cells uptake 18F-FLT and form an image [5, 22, 68]. In addition, 18F-FLT uptake needs the BBB as basis [28, 69]. So 18F-FLT-PET imaging is mainly based on cell proliferation, namely TK1 activity, and BBB permeability. In enhanced MRI, the imaging developing via gadolinium-DTPA (diethylenetriamine penta-acetic acid) (Gd-DTPA) is achieved by going through the BBB, which is associated with tumor vascularization and proliferation. Given the completeness of the BBB, when high-grade glioma patients have no significant 18F-FLT uptake, simultaneously, there would be no contrast enhancement in MRI [47]. Previous studies have shown that the reason why 18F-FLT uptake of normal brain tissue is not significant is the intactness of the BBB and lower proliferative activity, which in turn also confirmed the principle of glioma tissue uptaking 18F-FLT and imaging. 18F-FLT-PET has obvious advantages in actual clinical practice, which is the formation of a clear boundary between the glioma tissues and adjacent normal brain tissues. Of course, the destruction of the BBB has become a limitation of 18F-FLT-PET, in particular limiting its application in the diagnosis of low-grade gliomas [5].

Although 18F-FLT-PET imaging is based on the destruction of the BBB, when 18F-FLT-PET and conventional MRI were contrasted, previous study found that 18F-FLT-PET can reveal the extent of tumor that cannot be detected by enhanced MRI [28]. Zhao et al.’s study mentioned that the tumor boundary demonstrated by 18F-FLT-PET is not limited to which enhanced MRI showed. In most cases, it exceeds the scope of MRI can display. Furthermore, the expanding range of 18F-FLT uptake does not uniformly surround which MRI displays. Interestingly, in Yamamoto et al.’s Study, 18F-FLT accumulation volume is closely related to the enhanced amount of Gd-DTPA in enhancement MRI. Total tumor volume showed by 18F-FLT-PET is similar to that of enhanced MRI displays. However, the exact tumor boundaries showed by these two methods are not the same [84]. For postoperative residual tumor imaging of malignant glioma patients having received surgical treatment, 18F-FLT-PET does not necessarily show abnormalities consistent with postoperative MRI [86]. The real postoperative residual tumors may be undervalued or overvalued by MRI [82]. When cases having undergone resection of glioma receive conventional MRI and 18F-FLT-PET scan, if the cavity margin MRI displayed is larger than 18F-FLT-PET did, it might mean that the scope beyond is postoperative reaction and not the real residual tumor border.

### Various types of PET-CT in combination with conventional MRI

PET-CT scan of different tracer and conventional MRI each have their advantages and disadvantages. 18F-FDG-PET and 11C-MET-PET are currently the more widely used tests. The former has a great advantage for the identification of non-glioma tissue forming a good contrast with the non-18F-FDG-uptake necrotic tissue, while the latter can make the tumor and peritumoral tissues well distinguished in the image formed by PET-CT examination which will form relatively true and accurate tumor boundary. Furthermore, there is value for glioma grade prediction. Studies have shown that high-grade gliomas have a significantly higher 11C-MET, 18F-FDG uptake than low-grade gliomas. In evaluating the prognosis of glioma, 18F-FDG-PET has its prognostic value for glioma patients with a certain presence of contrast enhancement in enhanced MRI. But with the absence of contrast enhancement or in low-grade glioma patients, 11C-MET-PET can predict survival. In our review of relevant studies, 11C-MET-PET has a greater advantage than 18F-FDG-PET and MRI for low-grade glioma survival prediction. Moreover, 11C-MET-PET is not only stronger than conventional imaging in providing prognostic information, but also stronger than the histopathology. It is possible that tumor necrosis and the presence of BBB damage can lead to high uptake of 11C-MET. Therefore, 11C-MET-PET for high-grade gliomas provides no prognostic value. However, both methods have their own limitations. For example, due to normal tissue 18F-FDG uptake, 18F-FDG-PET fails to form the boundary between tumor tissue and normal brain tissue. The shorter half-life of 11C-MET limits its usage. Because of these limitations, 18F-FET-PET and 18F-FLT-PET emerged as more popular candidates. Comparing the two tests in the same patients, 18F-FET-PET can detect a larger tumor range than 18F-FLT-PET, indicating 18F-FET PET-CT can reflect the actual tumor boundary better. Furthermore, studies show that 18F-FET-PET has a higher detection rate of high-grade gliomas compared to 18F-FLT-PET. If the result of 18F-FET-PET is negative, then the possibility of coming to a diagnosis of malignant glioma is not high. 18F-FLT-PET imaging needs to be based on the BBB damage. Therefore, although some high-grade gliomas have a high proliferation index, 18F-FLT-PET probably remains of no use. Nevertheless, the boundaries shown by PET-CT remain the general boundaries of the tumor. For gliomas, especially malignant gliomas, there may be a single tumor cell or a plurality of tumor cells invading distantly. In this respect, it is difficult for the PET-CT to form an image. In terms of safety issues, there is no exact experimental evidence showing that PET-CT shares a long-term risk [3].

The notion that the application of CT and MRI can be used for distinguishing low-grade glioma tumor tissue from peritumoral normal brain tissue has already been recognized. With the continuous clinical practice, including enhancement MRI, conventional MRI scan alone has its limitations in identifying glioma as well as glioma grading. Biopsy or surgical resection decisions based solely on MRI information is unreliable. There may be false positive judgment of tumor grading and tumor invasion range, and this is explicitly presented in each study. For example, in T2-weighted FLAIR sequences exhibiting enhanced signal cases, only about half of the cases proved to be the actual tumor tissue after biopsy. There are also a considerable portion of patients with suspected glioma not presenting MRI contrast enhancement, or lack contrast-enhanced MRI at an early stage. However, the final pathologic diagnosis shows high-grade gliomas. Furthermore, for the follow-up evaluation of glioma patients having undergone multiple treatment, conventional MRI alone is not enough. At the same time, conventional MRI does have its indispensable role. Various studies have shown that conventional MRI combined with PET-CT is more valuable in judging tumor tissue and peritumoral brain tissue.

In clinical practice, advanced MRI is also valuable for the diagnosis of gliomas by providing more information on gliomas that is not available from conventional MRI. Examples of some available techniques include perfusion-weighted imaging (PWI), diffusion-weighted imaging (DWI), proton magnetic resonance spectroscopy (MRS), and diffusion tensor imaging (DTI) [41]. For instance, in PWI, the relative cerebral blood volume (rCBV) can be used to show malignant transformation of gliomas earlier than the emergence of new enhancement spot in enhanced MRI [1]. rCBV can be used to accurately differentiate grade II gliomas and grade III gliomas [87]. Higher-order diffusion techniques such as diffusion kurtosis imaging, a DWI-related new technology, can describe the changes in microstructure [31]. Preliminary studies suggest that this technique has a certain prospect in the differential diagnosis of brain tumors [31]. MRS can provide metabolic information of gliomas similar to that of PET-CTs or information complementary to PET-CTs. Based on the above, there are many existing studies that compare advanced MRI and PET and explore the significance of these two imaging methods combined in diagnosing glioma. In terms of DWI and PET-CT comparisons, studies have shown that DWI has limitations in diagnosing LGG. Compared with DWI, the diagnostic usage of 18F-FET-PET on LGG is irreplaceable [62]. In general, the accuracy of 1H MRS in diagnosing glioma progression was lower than that of 18F-FDG-PET [27]. 1H MRS has a higher diagnostic accuracy for LGG, and 18F-FDG-PET has a higher accuracy in the diagnosis of HGG [1, 27]. However, there are studies showing that MRS is valuable for the diagnosis of HGG [41]. This is a topic that is worth investigating in future research. On the other hand, advanced MRI and PET-CT can provide different information about gliomas. The information can enrich the understanding of gliomas before treatment. A study by Collet et al. suggests that advanced MRI and 18F-FLT PET-CT can improve the diagnostic efficiency of gliomas [10]. More prospective, large-sample experiments should be conducted in the future to explore methods to improve the accuracy of preoperative diagnosis of glioma using a combination of advanced MRI and PET-CT.

For neurosurgeons, it is important to have a precise selection of imaging examinations. For the patients admitted initially, conventional MRI is essential. It can provide anatomical information about glioma, which is the cornerstone of surgical treatment. PET-CT is of great importance for patients who have undergone MRI scan and been suspected suffering from gliomas, especially unclear-grade or high-grade gliomas. When concerning about the four different tracers of PET-CT, 18F-FDG PET-CT and 11C-MET PET-CT are two basic types of PET-CT scans. 18F-FDG PET-CT can show necrosis in tumor tissue. According to the different biological activity of gliomas, 11C-MET PET-CT can reflect the boundary of the tumor more closed to the biological situation, providing information for the glioma total resection. Both 18F-FDG PET-CT and 11C-MET PET-CT can give information on the grade prediction of gliomas based on the tracer uptake. In general, both 18F-FET PET-CT and 18F-FLT PET-CT are able to provide information for glioma border determinations. With the technique of multi-modality image fusion, PET-CT and MRI results can be fused to gain much more accurate information on the boundaries of gliomas, which can be utilized in the surgery (Table 3).

**Table 3:** Features of conventional MRI and different PET-CTs

	Imaging mechanism	Function	Characteristics	Shortcomings
Conventional MRI (including enhanced MRI)	Magnetic resonance	Showing the anatomical structure of glioma; providing preliminary information on the glioma boundary	Showing the anatomical structures clearly	The boundary of glioma shown is smaller than the biological boundary especially in higher grade glioma
18F-FDG PET-CT	Glucose metabolism of glioma cells	Showing the degree of metabolic activity of glioma; predicting the grade of glioma	Showing necrotic tissue	Normal brain tissue may also have high tracer uptake
11C-MET PET-CT	Amino acid metabolism of glioma cells	Showing glioma boundary; showing the degree of metabolic activity of glioma; predicting the grade of glioma	Showing a glioma boundary closer to the biological boundary	Relatively short half-life time
18F-FET PET-CT	Amino acid metabolism of glioma cells	Diagnosing glioma; predicting the grade of glioma	Relatively higher diagnosing accuracy of malignant glioma than conventional MRI	Blood-brain barrier damage leads to increased uptake
18F-FLT PET-CT	Nucleic acid metabolism of glioma cells	Showing glioma boundary	Showing a glioma boundary closer to the biological boundary than conventional MRI	Imaging depends on the blood-brain barrier damage; it is poor to show low-grade glioma

## Conclusion

Based on metabolic imaging, various PET-CTs showed different strengths but also different limitations. Overall, the studies, in varying degrees, were indicative of PET-CT to show tumor boundaries better than conventional MRI. But compared to PET-CT, conventional MRI can more clearly show the anatomical structure, which is a function cannot be replaced by any variety of PET-CT. Therefore, PET-CT and MRI are often combined to achieve high accuracy that any single examination method fails to achieve. In recent years, the concept of hybrid PET/MRI scanner is presented, fitting the conclusion of this systematic review and reflecting the new trend of glioma diagnosis. It can efficiently provide more comprehensive, high-resolution information for glioma preoperative planning, intraoperative neuro-navigation, and even postoperative treatments, such as radiotherapy [4].
